# Immunomodulatory Potential of *Tinospora cordifolia* and CpG ODN (TLR21 Agonist) against the Very Virulent, Infectious Bursal Disease Virus in SPF Chicks

**DOI:** 10.3390/vaccines7030106

**Published:** 2019-09-04

**Authors:** Swati Sachan, Kuldeep Dhama, Shyma K. Latheef, Hari Abdul Samad, Asok Kumar Mariappan, Palanivelu Munuswamy, Rajendra Singh, Karam Pal Singh, Yashpal Singh Malik, Raj Kumar Singh

**Affiliations:** 1 Immunology Section, ICAR-Indian Veterinary Research Institute, Izatnagar, Bareilly 243 122, Uttar Pradesh, India; 2 Division of Pathology, ICAR-Indian Veterinary Research Institute, Izatnagar, Bareilly 243 122, Uttar Pradesh, India; 3 Division of Physiology and Climatology, ICAR-Indian Veterinary Research Institute, Izatnagar, Bareilly 243 122, Uttar Pradesh, India; 4 Division of Biological Standardization, ICAR-Indian Veterinary Research Institute, Izatnagar, Bareilly 243 122, Uttar Pradesh, India; 5 ICAR-Indian Veterinary Research Institute, Izatnagar, Bareilly 243122, Uttar Pradesh, India

**Keywords:** IBDV, *Tinospora cordifolia*, TLR agonist, CpG ODN, immunomodulator, adjuvant

## Abstract

Infectious bursal disease (IBD), caused by infectious bursal disease virus (IBDV), is characterized by severe immunosuppression in young chicks of 3 to 6 week age group. Although vaccines are available to prevent IBD, outbreaks of disease are still noticed in the field among vaccinated flocks. Further, the birds surviving IBD become susceptible to secondary infections caused by various viral and bacterial agents. This study assessed the immunoprophylactic potential of Cytosine-guanosinedeoxynucleotide (CpG) oligodeoxynucleotides (ODN) and *Tinospora cordifolia* stem aqueous extract in the specific pathogen free (SPF) chicks, experimentally infected with very virulent IBDV (vvIBDV). Both of these agents (CpG ODN and herbal extract) showed significant increase in the IFN-γ, IL-2, IL-4, and IL-1 levels in the peripheral blood mononuclear cells (PBMCs) (*p* < 0.05) of chickens in the treatment groups following IBD infection. Further we found significant reduction in mortality rate in vvIBDV infected chicks treated with either, or in combination, compared with the birds of control group. Additionally, the adjuvant or immune enhancing potential of these two immunomodulatory agents with the commercially available IBDV vaccine was determined in chicks. The augmentation of vaccine response in terms of an enhanced antibody titer after vaccination, along with either or a combination of the two agents was noticed. The findings provide a way forward to counter the menace of IBDV in the poultry sector through use of these herbal or synthetic immunomodulatory supplements.

## 1. Introduction

Infectious bursal disease (IBD), caused by IBD virus (IBDV), is an acute, highly contagious, and immunosuppressive disease particularly affecting chicks of 3 to 6 weeks age worldwide. The disease results in severe economic loss to poultry farmers. IBDV is a non-enveloped, double stranded RNA virus belonging to the genus *Avibirnavirus* of the family *Birnaviridae* [1]. This virus primarily inflicts cytolysis of dividing cells in primary lymphoid organ, bursa of Fabricius (BF) in chicks, leading to severe immunosuppression [2], and fatal complications, such as high mortality, poor weight gain, and condemnation of the carcasses because of hemorrhages in the skeletal muscles of IBD affected chickens [3]. Transmission of IBDV is via the fecal-oral route. Out of two serotypes of IBDV, serotype 1 is pathogenic, while serotype 2 is non-pathogenic. Serotype 1 is further classified into classical, intermediate, and very virulent strains [4]. IBDV infection prevails in vaccinated broiler stock in Indian states throughout the year with the highest incidence in monsoon season (July to October) [5]. Although live attenuated vaccines are available to prevent IBD, field outbreaks are not uncommon in vaccinated flocks, which might be due to the emergence of antigenic variants of IBDV in the field settings [6]. Besides the direct consequences, IBD outbreak also lead to the vaccination failures of other diseases in the recovered birds, due to immunosuppression [7], thus extending the negative impact of IBD over poultry sector.

Augmentation of innate immune responses through immunomodulatory supplements may help to overcome such challenges in the management of IBDV induced infection and associated secondary complications. Amid several agents known to augment the innate immune response, Toll like receptors’ (TLR) agonists and some herbs can further improvise an appropriate adaptive immune response, countering the invading pathogen efficiently [8,9]. TLRs, the key sensors of innate immunity, are evolutionary conserved pattern recognition receptors (PRRs) found in both mammals and avian species [10]. They recognize conserved structural motifs on various pathogens, termed pathogen associated molecular patterns (PAMPs). Recognition of PAMPs by TLRs on immune cells leads to activation of the signaling pathways, and provokes cellular activation and release of cytokines [11]. This further activates the adaptive immune system, due to the maturation of antigen presenting cells. Owing to said properties, the TLR ligands can be employed either as prophylactic agents or as vaccine adjuvants against various pathogens [12,13,14]. The chicken’s TLR repertoire consists of 10 TLRs; viz., TLR1LA, TLR1LB, TLR2A, TLR2B, TLR3, TLR4, TLR5, TLR7, TLR15, and TLR21 [15]. Avian TLR21 is a cytoplasmic receptor which recognizes microbial DNA (containing unique CpG dinucleotide motifs) as a danger signal, similarly to typical TLR9 in mammalian species. Recognition of CpG DNA by TLR21 induces activation of NF-ĸB pathway, leading to up regulation of pro-inflammatory molecules, including nitric oxide (NO), and recruitment of innate immune cells [16]. Such activation of TLR21 has shown an antiviral effect against viruses, such as against the avian influenza virus (AIV) in birds [17]. CpG ODN has also shown promising results to enhance the efficacy of the IBDV DNA vaccine and attenuated vaccine, compared to the use of the live vaccine used alone [18,19].

Herbs have been used widely in India under the ayurvedic system of medicine since time immemorial. Several studies have proven the immunomodulatory potential of various herbs; to name few, *Astragalus sp*, *Withania somnifera*, *Tinospora cordifolia*, *Azadirachta indica*, and *Phyllanthus emblica*. [20,21,22]. Amongst them, *T. cordifolia* (guduchi, common name) has been well established for potent immunomodulatory properties [23,24]. Guduchi aqueous extract has been shown to activate macrophages, which form the first line of defense against pathogens invading the living system [25]. Guduchi has shown an antiviral effect against chicken infectious anemia virus (CIAV) infection in poultry, along with an immunomodulatory effect to overcome immunosuppression caused by this virus [21].

In this study, we established the interaction of CpG ODN (TLR21 agonist) and guduchi aqueous extract in providing protection against IBDV, and their mechanism of interaction. Furthermore, adjuvant action of CpG ODN and guduchi aqueous extract with commercially available IBDV vaccine was also evaluated. To the best of our knowledge, this is the first study demonstrating the interaction of any TLR agonist and herbal extract for providing protection against a viral infection in chicken.

## 2. Materials and Methods

### 2.1. Experimental Animals

Specific pathogen free (SPF) eggs (*n* = 170) were procured from Venky’s Pvt. Ltd., (Pune, India) and hatched at the Central Avian Research Institute (CARI), Izatnagar. The chicks were maintained at the experimental animal shed facility of the Avian Disease Section, ICAR-IVRI, Izatnagar, maintaining required pathogen free settings. The experiment had prior approval from the institute animal ethics committee (number F. 26-1/2015-16/JD(R)).

### 2.2. Virus

A very virulent strain of IBDV (vvIBDV) (GenBank accession: KM205793.1 (VP2 gene)), maintained in the viral laboratory of the Avian Disease Section, was used for the experimental infection after passaging in three-week-old SPF White Leghorn (WL) chicken. At 72 h post-inoculation, the bursae were harvested, chopped, and homogenized in peptone broth containing penicillin and streptomycin (1000 mg/mL each). The filtered supernatant obtained from the bursal homogenate was used as a source of the vvIBDV for further work. The virus suspension was titrated in 10 day-old SPF embryonated chicken eggs by inoculation into the chorioallantoic membrane route [26].

### 2.3. TLR21 Agonist

Chicken TLR21 agonist (CpG ODN) was procured from Alpha Diagnostic Intl Inc, San Antonio, TX, USA. (Cat. #ODN2007-1), and was used in the study after making appropriate dilutions.

### 2.4. Preparation of T. Cordifolia Aqueous Extract

*T. cordifolia* stem powder was procured commercially (Herbal Hills, Mumbai, India) and was used for preparing an aqueous extract for further use in the experiment. Aqueous extract was prepared by boiling stem powder of this plant in water at a 1:5 ratio, at 100 °C for 30 min [27], followed by filtration of the mixture. Filtrate was stored in refrigerator until further use.

### 2.5. Vaccines

Commercially available IBDV (Intermediate and Intermediate Plus) vaccines (Venky’s India Pvt. Ltd., Pune, India) and Newcastle disease virus (NDV) vaccine, lentogenic ‘F’ strain (Biomed Pvt. Ltd., Ghaziabad, India) were used in the present study.

### 2.6. ELISA Kit

The ELISA kit for IBDV was procured from IDEXX Laboratories, Westbrook, MA, USA.

### 2.7. Primers

The published oligonucleotide primers selected for chicken specific genes; viz., β-actin, IL-2, IFN-γ, IL-4, IL-1β, and vvIBD-VP2 (Table 1), were used in the study. All primers were synthesized from Eurofins Genomics India Pvt. Ltd. (Bangalore, India).

### 2.8. Experiment 1

A total of 100 SPF WL birds, three-weeks old, were randomly divided into five groups; viz. A, B, C, D, and E (*n* = 20/group). Group A was kept as a negative control and was given water in a pulsating manner (over a period of eight hours), 10 mL/bird/day orally, to mimic the *T. cordifolia* extract treatment (control for group C). Group B (positive virus control) was given phosphate buffered saline (PBS, pH 7.2) via intramuscular route at 4 weeks of age. Birds in group C were given *T. cordifolia* aqueous extract from day one until 4 weeks of age in a pulsating manner (over a period of eight hours), 10 mL/bird/day orally. Group D was given CpG ODN, 50 µg/bird via intramuscular injection at 4 weeks of age. Group E was given both aqueous extract, prepared as above, from one day old, until 4 weeks of age, in a pulsating manner, 10 mL/bird/day; and CpG ODN, 50 µg/bird via intramuscular route at 4 weeks of age. Birds in group B, C, D, and E were challenged with 10^5^ ELD_50_ of vvIBDV at 24 h post-injection of CpG ODN through the ocular route and observed for the clinical signs and mortality until 10 days post-challenge.

#### 2.8.1. Collection of Samples

Blood samples (*n* = 6/group) were collected in heparinized vials (20 IU/mL) at the 4th (before challenge), 5th (7 days post challenge (DPC)), and 6th (14 DPC) week of age. On day four post-challenge, three birds from each group (A–E) were sacrificed for the collection of bursa. A piece of bursal tissue was stored in 10% neutral buffered formalin for histopathology (HP), and the remaining half of the bursal tissue was stored in an RNA *later* (Qiagen India Pvt. Ltd., New Delhi, India) solution at −80 °C for cytokine expression studies.

#### 2.8.2. Isolation of Chicken Peripheral Blood Mononuclear Cells (PBMCs)

PBMCs were isolated from the blood, as described previously [30]. Briefly, blood was layered over an equal volume of Ficoll Hypaque (Sigma Chemical Co., St. Louis, MO, USA) with a density of 1.077 g/mL and kept for centrifugation at 500× *g* for 45 min for phase separation. The interface layer containing the PBMCs was aspirated carefully and washed in sterile PBS twice, and finally cell pellet was suspended in 1 mL sterile PBS. Cell viability was checked by trypan blue dye exclusion method, and around 1 × 10^6^ cells from each sample were stored in a 1 mL RiboZol (Genetix Biotech, New Delhi, India) at −80 °C until RNA extraction.

#### 2.8.3. RNA Extraction and cDNA Synthesis

Total RNA was extracted from the PBMCs and bursal tissue, using RiboZol reagent as per manufacturer’s guidelines. Briefly, bursal tissue was homogenized in RiboZol (1:10) and 250 µL PBMCs mixed with 750 µL RiboZol, followed by addition of chloroform (200 µL) and centrifuged at 12,000 rpm for 15 min at 4 °C for phase separation. Aqueous phases were precipitated with 500 µL isopropanol followed by centrifugation (12,000 rpm for 15 min at 4 °C), supernatants were removed, and the RNA pellets were washed carefully with 75% ethanol and centrifuged (7500 rpm for 5 min at 4 °C). After removing ethanol, RNA pellets were dissolved in RNase free water, warmed at 55–60 °C and then stored at −20 °C, until further use. RNA purity and quantity were determined by Nano-drop spectrophotometer analyzer (Thermo Fischer Scientific, Waltham, MA, USA) at A260/280.

Reverse transcription (RT) for the first strand synthesis was carried out by using Revert-Aid Reverse Transcriptase (Thermo Scientific, Waltham, MA, USA) in a standard 20 µL reaction mixture. To a sterile nuclease free 0.2 mL PCR tube, the reaction mixture was prepared using: RNA template (1 μg), Random Hexamer Primer 100 µM (0.2 μg/μL, 1.0 μL), dNTP mix (10 mM each) (2.0 μL) and nuclease free water (NFW) (to make up to 20 µL volume). The mixture was incubated at 70 °C for 5 min, followed by immediate snap chilling in ice. Then reagents were added as follows, 5X RT buffer (4.0 μL), RiboLock RNase inhibitor (40 U/μL, 0.5 μl) and RevertAid Reverse Transcriptase (200 U/μL, 1.0 μL). RNA was subsequently reverse-transcribed at 25 °C for 10 min, and 42 °C for 60 min, followed by 10 min at 70 °C to denature the enzyme. All these steps were carried out in a thermal cycler (QB 96 Server Gradient Thermal Cycler, Quanta Biotech Ltd., Surrey, UK). The cDNA samples were cooled at 4 °C and stored at −20 °C.

#### 2.8.4. Quantitative Real-Time PCR

SYBR green-based quantitative real-time PCR was performed using Stratagene MX300 5P for studying the expression of different immune response genes. β-actin was used as the internal control gene. Each sample was run in duplicate and the total reaction volume was 20 µL, containing SYBR green master mix (Thermo Fischer Scientific, Lithuania, Waltham, MA, USA), 10 µL, template cDNA, 1 µL, gene specific forward and reverse primers, 0.5 µL each, and NFW, 8 µL. Real-time PCR conditions were one cycle at 95 °C for 10 min, 40 cycles of 95 °C for 15 s, 60 °C for 15 s, 72 °C for 15 s and one cycle 95 °C for 1 min; and a 65–95 °C ramp for melt curve analysis to check the amplicon specificity.

#### 2.8.5. Evaluation of the Humoral Immune Response Using ELISA

The humoral immune response against vvIBDV was assessed using an ELISA kit (IDEXX Laboratories, Westbrook, ME, USA) following the manufacturer’s protocol on collected sera samples. Briefly, antigen coated plates were taken, and sample positions were recorded, followed by the addition of 100 µL of undiluted negative control (NC), 100 µL of undiluted positive control (PC), and 100 µL of respective diluted samples (1:500) into appropriate wells. Plates were incubated for 30 min at 18–26 °C, followed by removal of the solution in each well and thrice washing with distilled water (350 µL/well). 100 µL of conjugate was dispensed into each well, incubated for 30 min at 18–26 °C, and followed by washing, as previously explained. 100 µL of TMB substrate was dispensed into each well, incubated for 15 min at 18–26 °C and finally 100 µL of stop solution was dispensed. Absorbance was recorded at 650 nm using the ELISA reader.

The antibody titer was calculated as per the equations provided in the ELISA kit;

Controls

$${\mathrm{NC}}_{\mathrm{mean}}=\frac{\mathrm{NC}1\;A\left(650\right)+\mathrm{NC}2\;A\left(650\right)}{2}$$

$${\mathrm{PC}}_{\mathrm{mean}}=\frac{\mathrm{PC}1\;A\left(650\right)+\mathrm{PC}2\;A\left(650\right)}{2}$$

Samples

$$S/P=\mathrm{sample}\;\mathrm{mean}-{\mathrm{NC}}_{\mathrm{mean}}/{\mathrm{PC}}_{\mathrm{mean}}-{\mathrm{NC}}_{\mathrm{mean}}$$

where, S/P = sample to positive ratio

$${\mathrm{Log}}_{10}\mathrm{Titre}=1.09\left({\mathrm{Log}}_{10}S/P\right)+3.36$$

Antibody titer is the antilog of that value.

#### 2.8.6. Protection Study against the vvIBDV Challenge

The protection study included observation of clinical signs and mortality up to ten days post-challenge, viral load estimation, and gross and histopathological examination of bursae.

##### Gross and Histopathological Evaluation

Necropsy was done on dead birds to record the gross lesion and post-mortem diagnosis. Bursae from dead as well as sacrificed birds were processed for routine paraffin embedding technique. Tissue sections of 4–5 mm thickness were stained with hematoxylin and eosin (H&E) for microscopic evaluation. Bursal lesions (BLS) were scored on a scale of 0 to 5 (0: no lesion, 1: slight change, 2: scattered or partial bursal damage, 3: 50% or less follicle damage, 4: 51–75% follicle damage, and 5: 76–100% bursal damage) by a trained avian pathologist, following the blind folded method, as described elsewhere [31].

##### Viral Load Estimation

RNA samples from bursal tissues collected at 4 DPC of vvIBDV infection from all the groups was used for cDNA synthesis, followed by quantitative real-time PCR for IBDV specific gene (VP2). Real-time PCR conditions included one cycle at 95 °C for 8 min, 40 cycles of 94 °C for 1 min, 57 °C for 50 s, 72 °C for 50 s, and one cycle of 95 °C for 1 min; and 65–95 °C for melt curve analysis to check the amplicon specificity. For evaluating the concentrations of virus in bursal samples, a standard curve was prepared for the VP2 gene amplified in the pDrive Cloning Vector.

#### 2.8.7. Assessment of Protection against NDV

On day 10 post-vvIBDV challenge, the surviving birds were vaccinated with Newcastle disease virus (NDV) vaccine, lentogenic ‘F’ strain (Biomed Pvt. Ltd., Ghaziabad, India) through the ocular route with the recommended dose. Sera were collected at weekly intervals (*n* = 5/group) for three weeks post-vaccination, and stored at −20 °C until further use.

#### 2.8.8. Evaluation of the Humoral Immune Response against the NDV Vaccine

The humoral immune response against the NDV vaccine was measured by hemagglutination inhibition (HI) test, according to the OIE-World organization for animal health recommended protocol [32], using antigen prepared from the lentogenic strain (F) of NDV and 1% chicken RBCs.

### 2.9. Experiment 2

*The evaluation of the immune-enhancing effect of T. cordifolia and a TLR21 agonist (CpG ODN) on IBDV vaccine responses in chicks.*

Fifty SPF WL birds of two-week age were randomly divided into five groups; viz. A, B, C, D, and E (*n* = 10/group). Group A was kept as a negative control. Group B was kept as a positive control. Birds in group C were given *T. cordifolia* aqueous extract from day one until 4 weeks of age in a pulsating manner (over a period of eight hours), 10 mL/bird/day orally. Group D was given CpG ODN, 10 µg/bird via the intramuscular route at 2 weeks, repeated at 4 weeks of age. Group E was given both aqueous extract, prepared as above, from day one until 4 weeks of age in a pulsating manner (over a period of eight hours), 10 mL/bird/day; and CpG ODN, 10 µg/bird via the intramuscular route at 2 weeks, and again at 4 weeks of age. Chicks in group B to E were vaccinated with IBDV intermediate (live attenuated, each dose of vaccine containing ≥ 10^2^ EID_50_ of IBD Intermediate standard strain) vaccine at 2 weeks and a booster dose at 4 weeks of age, with IBDV intermediate plus vaccine (each dose of vaccine containing Intermediate Plus strain ≥ 10^2^ EID_50_), while group A acted as negative control.

Blood samples without anticoagulant (*n* = 6/group) were collected from birds in all the groups pre-vaccination; and at 7, 14, 21, and 28 days post-vaccination (DPV) in vaccinated birds, for assessing the IBDV specific antibody responses. IBDV specific antibody responses were measured using a commercially available ELISA kit (IDEXX), as described in the earlier procedure.

### 2.10. Data Analysis

Relative expressions in target genes were analyzed based on the 2−^ΔΔCT^ method, previously described by Pfaffl, 2001 [33], normalizing against the β-actin gene and expressing the outcome as fold change over the control gene. All the data are expressed as mean ± SEM. All the results were analyzed using the statistical software GraphPad Prism version 8.1.2. Two-way analysis of variance (ANOVA) was used to determine the statistically significant differences in mean values between the groups (except immune response genes in PBMCs ex vivo, which were analyzed using the ‘*t*’ test). The control group was considered a calibrator for relative quantification of mRNA expression. Multiple comparisons between groups were done through Tukey’s post hoc test. Values of *p* < 0.05 were considered significant.

## 3. Results

### 3.1. Immune Response Genes in PBMCs Ex Vivo

**Figure 1:**
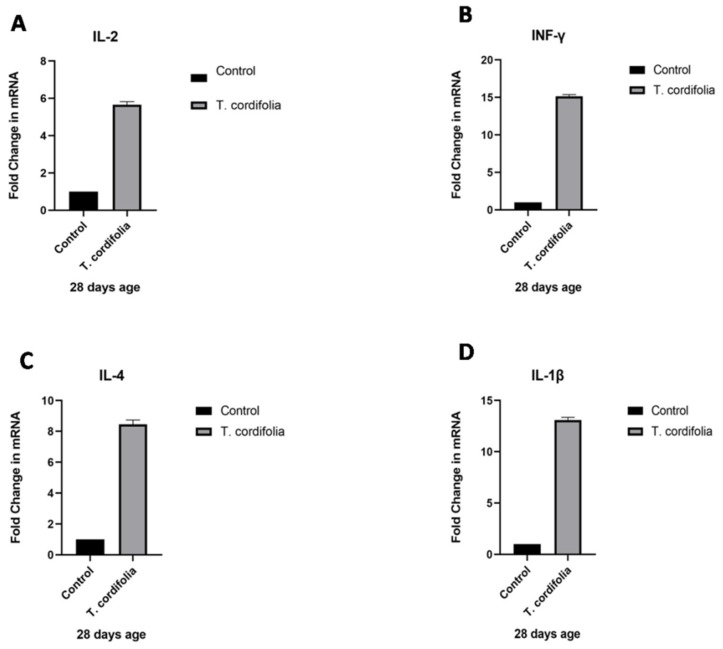
(**A**–**D**) Quantitative real-time PCR analysis of Immune response gene transcripts (expressed as fold change in the immune response gene transcripts) in PBMCs ex vivo in specific pathogen free (SPF) WL chickens post *T. cordifolia* treatment for 4 weeks.

The guduchi treated (after oral administration of *T. cordifolia* aqueous extract to birds at 10 mL/day/bird from one day old to 4 weeks of age) group showed significant increases (*p* < 0.05) in IL-2 (5.657 ± 0.1663), IFN-γ (15.15 ± 0.2288), IL-4 (8.458 ± 0.277), and IL-1β (13.09 ± 0.2681) expressions in chicken PBMCs compared to the control group (Table 2, Figure 1A–D). Though a significant increase in the immune response gene expression is observed, there were still no clinical symptoms of illness observed in treated chicks, thereby suggesting that *T. cordifolia* extract usage is apparently safer in chicks without any autoimmunity or immunopathological changes

**Figure 2:**
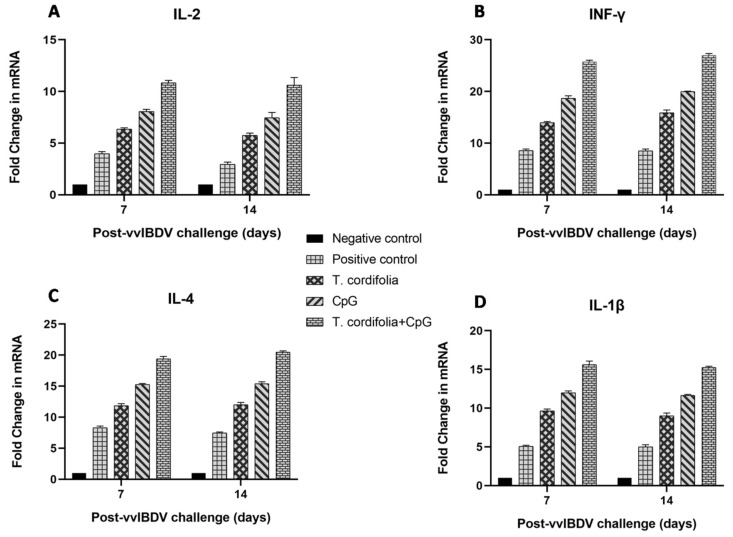
(**A**–**D**). Quantitative real-time PCR analysis of immune response gene transcripts in PBMCs ex vivo in SPF WL chickens 7 and 14 days post-vvIBDV challenge. The result is expressed as fold change in the immune response gene transcripts relative to the control group.

The group E showed an increase (*p* < 0.05) in IL-2 expression in PBMCs at 7 DPC (10.840 ± 0.232) and 14 DPC (10.620 ± 0.723) which was the only increase, and was followed by group D, which showed significant differences from groups A, B, and C at 7 DPC (8.068 ± 0.207) and 14 DPC (7.462 ± 0.507) (Table 3, Figure 2A).

The group E showed an increase (*p* < 0.05) in IFN-γ expression in PBMCs at 7 DPC (25.702 ± 0.328) and 14 DPC (26.982 ± 0.345) which was the only increase, and was followed by group D, which showed significant differences from groups A, B, or C at 7 DPC (18.672 ± 0.490) and 14 DPC (19.973 ± 0.094) (Table 4, Figure 2B).

The group E showed an increase *(p* < 0.05) in IL-4 expression in PBMCs at 7 DPC (19.400 ± 0.376) and 14 DPC (20.468 ± 0.207). Group D, followed, but showed significant differences from groups A, B, and C at 7 DPC (15.265 ± 0.162) and 14 DPC (15.397 ± 0.293) (Table 5, Figure 2C).

The group E showed an increase (*p* < 0.05) in IL-1β expression in PBMCs at 7 DPC (15.628 ± 0.444) and 14 DPC (15.247 ± 0.169) which was the only increase, followed by group D which showed significant differences from groups A, B, and C at 7 DPC (11.987 ± 0.243) and 14 DPC (11.660 ± 0.123) (Table 6, Figure 2D).

### 3.2. Immune Response Genes in the bursa of Fabricius Ex Vivo

**Figure 3:**
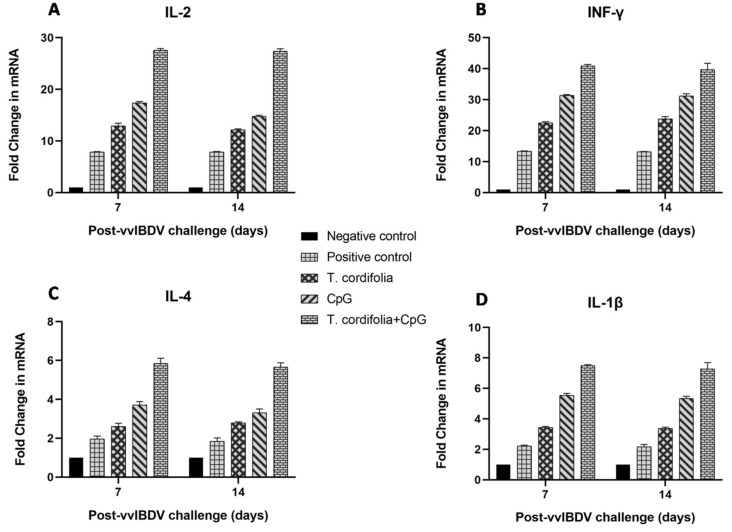
(**A**–**D**). Quantitative real-time PCR analysis of immune response gene transcripts in the bursa of Fabricius ex vivo, in SPF WL chickens 7 and 14 days post-vvIBDV challenge. The results are expressed as fold changes to the immune response gene transcripts relative to the control group.

The group E showed an increase (*p* < 0.05) in IL-2 expression in PBMCs at 7 DPC (27.550 ± 0.363) and 14 DPC (27.370 ± 0.496), which was followed by group D, which showed significant difference from group A, B and C at 7 DPC (17.397 ± 0.232) and 14 DPC (14.797 ± 0.189) (Table 7, Figure 3A).

Group E showed an increase (*p* < 0.05) in IFN-γ expression in PBMCs at 7 DPC (40.892 ± 0.446) and 14 DPC (39.693 ± 2.031) which was the only increase, followed by group D, which showed significant difference from group A, B and C at 7 DPC (31.348 ± 0.298) and 14 DPC (31.232 ± 0.659) (Table 8, Figure 3B).

Group E showed an increase (*p* < 0.05) in IL-4 expression in PBMCs at 7 DPC (5.842 ± 0.272) and 14 DPC (5.663 ± 0.211) which was the only increase, followed by group D, which showed significant differences from groups A, B, and C at 7 DPC (3.712 ± 0.172) and 14 DPC (3.310 ± 0.195) (Table 9, Figure 3C).

Group E showed an increase (*p* < 0.05) in IL-1β expression in PBMCs at 7 DPC (7.498 ± 0.054) and 14 DPC (7.275 ± 0.413) which was the only increase, followed by group D, which showed significant differences from groups A, B, and C at 7 DPC (5.543 ± 0.128) and 14 DPC (5.345 ± 0.133) (Table 10, Figure 3D).

### 3.3. The Humoral Immune Response (vvIBDV Post Challenge, Based on an Antibody Titer Estimated Using an ELISA Kit)

**Figure 4:**
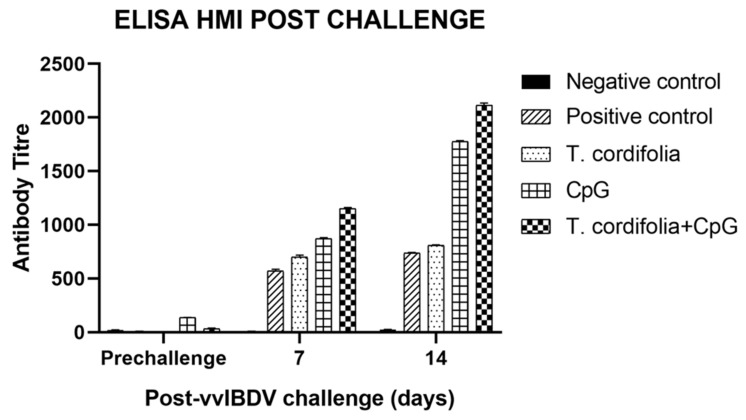
Serum antibody titer in chickens challenged with vvIBDV at different DPC by ELISA kit method (antibody titer is expressed as mean ± SE of antilog of Log_10_ titer).

Group E showed a significant difference (*p* < 0.05) from other groups at 7 DPC (1152.477 ± 8.040) and 14 DPC (2115.643 ± 18.462), with the maximum antibody titer being estimated in this group. Following E, was group D, which differed significantly (*p* < 0.01) at 7DPC (871.597 ± 7.625) and 14 DPC (1776.421 ± 7.847) from group C and group B. Additionally, in both group D and group E antibody titer significantly increased (*p* < 0.01) from 7 DPC to 14 DPC. Group C also showed a significant increase (*p* < 0.01) in antibody titer 7 DPC (699.968 ± 17.517) and 14 DPC (809.135 ± 3.712) from the positive virus control (group B) (Table 11, Figure 4).

### 3.4. Protection against vvIBDV Challenge

**Figure 5:**
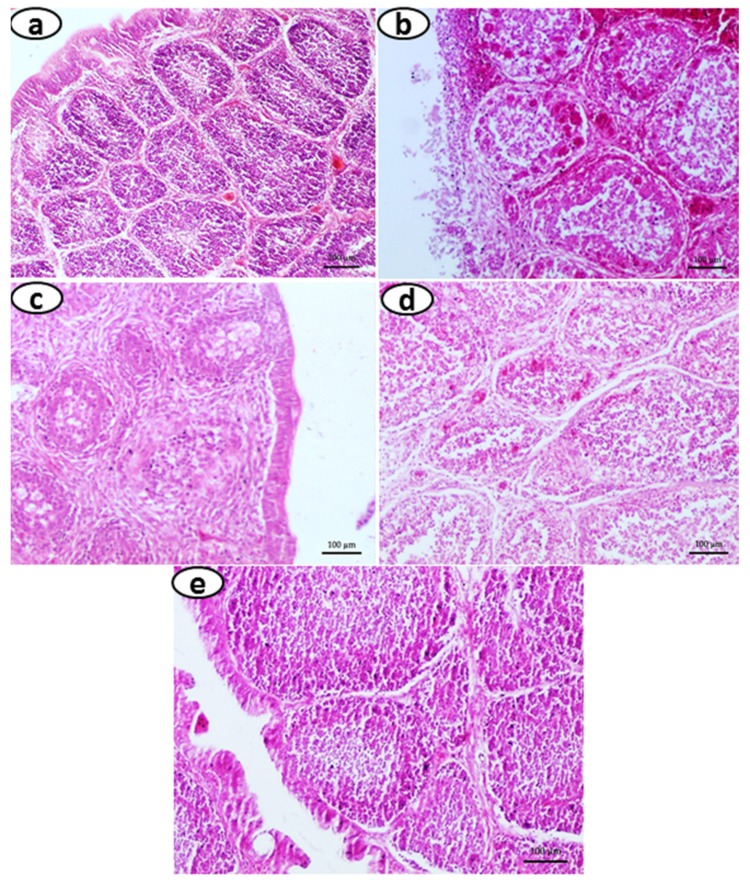
Section of bursa of Fabricius showing (**a**) Normal histo-architecture characterized by bursal follicles with intact cortex and medulla, along with lining epithelium; (**b**) Bursal follicles with severe lymphoid depletion in the cortex and medulla, severe congestion, and hemorrhages are evident. Loss of plical lining epithelium is seen; (**c**) moderate to severe lymphoid depletion in the bursal follicles, along with normal lining of plical epithelium and peri-follicular fibrosis is evident; (**d**) moderate lymphoid depletion in the bursal follicles along with mild peri-follicular fibrosis; (**e**) apparently normal histo-architecture, characterized by bursal follicles with intact cortexes and mild lymphoid depletion in the medulla, along with intact lining epithelium (H&E 20X).

**Figure 6:**
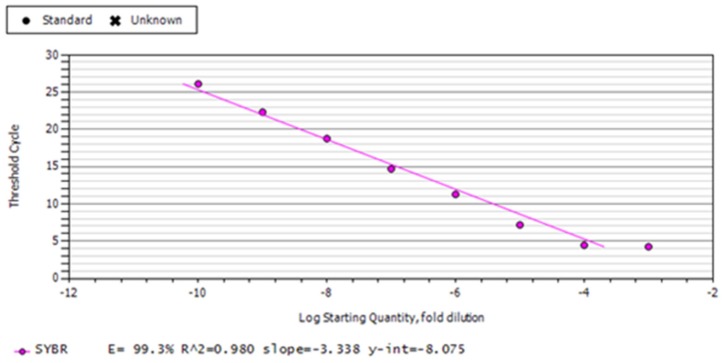
Standard curve with VP2 gene (189 bp) cloned in a pDrive cloning vector for viral load estimation.

(a) *Clinical signs and mortality:* No clinical signs and mortality were observed in any of the birds for the first 24 h post challenge with vvIBDV. After 48 h, affected birds showed clinical signs, such as drowsiness, depression, ruffled feathers, inability to move, and severe prostration. Group E showed the highest protection (100%), followed by group D (80%), and group C (50%), whereas 100% mortality was observed in group B (Table 12).
(b) *Gross lesions and Histopathology:* In group A (negative control), the bursa of Fabricius revealed apparently normal histo-architecture, characterized by intact lining epithelia, lymphoid follicles with sufficient numbers of lymphocytes in the cortex, and medulla with thin connective tissue stroma separating each follicle (Figure 5a). No pathological lesions could be detected in the bursa of Fabricius of the control group. In group B (positive virus control), the lesions in the bursa of Fabricius included moderate to severe hemorrhages, edema, and partial to complete destruction of bursal follicles, with or without perifollicular fibrosis. Severe lymphocytolysis with marked perifollicular fibroplasia was also observed in bursa of Fabricius (Figure 5b). In group C, there were hemorrhages, and there was mild to moderate lymphocytolysis, marked reticuloendothelial cell hyperplasia, and congestion in stromal blood vessels (Figure 5c). In group D, the bursa of Fabricius exhibited very little histopathological alterations, characterized by mild peri-follicular edema and reticuloendothelial cell hyperplasia (Figure 5d). In group E, the histo-architecture of the bursa of Fabricius was intact. No pathologically significant alterations in the stromal connective tissue were observed (Figure 5e). But in 20% of the birds, a mild degree of edema in the bursal follicles and only mild depletion of lymphocytes in the medulla were observed.
(c) *Viral load:* Real time quantification of viral load from bursa of Fabricius in birds from groups A and C to E was done by using VP2 as the target gene, cloned in a pDrive Cloning Vector (not done in birds from group B as none of those survived the virus challenge). The viral copy number of samples was calculated by interpolating the samples’ Ct values with Ct value of serially diluted VP2 cloned plasmid. The group E showed the lowest (*p* < 0.05) of copy number of all the groups at 4 DPC (2.647 ± 0.277), followed by the group D (3.977 ± 0.222), and then by group C (5.910 ± 0.262). The highest copy number was found in group A (8.840 ± 0.180) (Table 13, Figure 6).
(d) *Humoral immune response analysis after NDV vaccination:* The birds treated with both *T. cordifolia* and CpG ODN (group E) prior to the vvIBDV challenge showed a highly significant increase in HI titer, more so than those of groups C and D at two and three weeks post-NDV vaccination (*p* < 0.05) (Table 14). The antibody titer in group A and E was almost similar at two and three weeks post-NDV vaccination. However, no significant difference was noticed at one week post-NDV vaccination among all the groups.

### 3.5. The Assessment of the Humoral Immune Response after Vaccination by ELISA Kit

**Figure 7:**
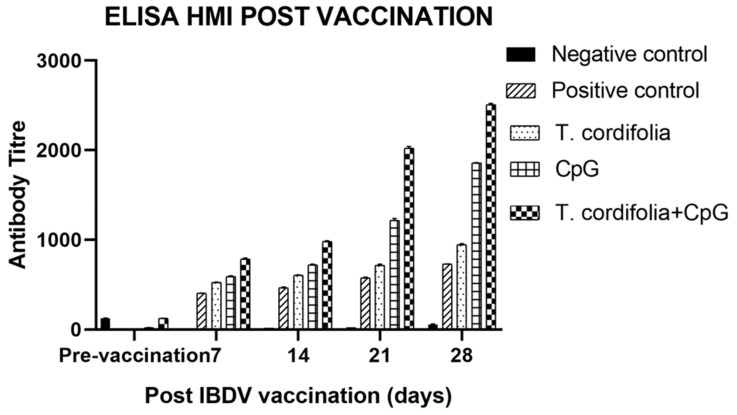
Serum antibody titer in chickens vaccinated with IBDV at different DPV by ELISA kit method. Antibody titer is expressed as mean ± SE of antilog of Log_10_ titer.

The results of ELISA for serum antibody titer estimation after IBDV vaccination are shown in Table 15 and Figure 7.

The group E showed a significant difference (*p* < 0.05) from other groups at 7 DPV (784.827 ± 12.559), 14 DPV (979.217 ± 9.894), 21 DPV (2020.827 ± 20.893), and 28 DPV (2509.359 ± 12.774), with the maximum antibody titer being estimated in this group; followed by the group D which differed significantly (*p* < 0.01) at 7DPV (587.897 ± 11.084), 14 DPV (717.898 ± 11.729), 21 DPV (1217.303 ± 18.584), and 28 DPV (1857.785 ± 4.094) from group C and group B. Furthermore, in both group D and group E, antibody titer significantly increased (*p* < 0.01) from 7 DPV to 28 DPV. Group C also showed a significant increase (*p* < 0.01) in antibody titer at 7 DPV (525.834 ± 3.913), 14 DPV (602.523 ± 5.427), 21 DPV (716.618 ± 11.656), and 28 DPV (944.575 ± 12.222), from group B.

## 4. Discussion

Infectious bursal disease virus induced immunosuppressive disease is one of the serious hurdles faced by commercial poultry producers throughout the world. This virus causes severe acute infection in young chickens, affecting mainly the bursa of Fabricius, leading to death of lymphoid cells [34]. IBDV is ubiquitous, and most often chickens acquire infection via the oral route. Viral replication occurs in bursa of Fabricius, and IgM bearing B cells are the target of this virus [35]. Immunosuppression induced by this virus affects chickens in serious ways; viz., increased incidence of secondary infections, poor feed conversion, reduction in protective immune response generated by other vaccines, etc. [19]. Though vaccination is imperative in preventing the incidences of infectious diseases, at certain times this procedure fails in the field. Similarly, vaccines available to protect IBDV challenge are not fully protective, despite the high antibody titer generated by them [36]. Live vaccines available induce bursal atrophy, along with possessing unstable antigenic and pathogenic characteristics [34]. This leads to increase in susceptibility to other secondary bacterial infections [37]. Thus, raising the need to find immunomodulatory agents which may act as standalone armor to combat such pathogens, or when used with vaccines, to increase their effectiveness in the host.

For time immemorial, herbs have been used in India traditionally for their immunomodulatory and prophylactic potential against various pathogens. *T. cordifolia* is one such herb with immense immunomodulatory potential in animals and poultry [21,24]. Previous studies on *T. cordifolia* supplementation in broilers indicated its use as an alternative to an antibiotic growth promoter and immunostimulator, showing positive impacts on feed conversion ratio and overall growth improvement without any negative effects or toxicity [38,39]. Similarly, in the present study, *T. cordifolia* extract feeding for chicks up to 4 weeks of age does not show any observable ill effects. Thus, its use was found safe for chickens, without causing any harm to them with autoimmunity or immunopathology due to the stimulation of immune system.

In this study, the aqueous extract of *T. cordifolia* stem powder was given from one day old until 4 weeks of age. Expressions of IL-2, IFN-γ, IL-4, and IL-1β were estimated in chicken PBMCs at 4 weeks of age. The results indicated significant up-regulation of these cytokine genes in PBMCs isolated from supplemented birds, compared with the control. These results are in accordance with the study where polysaccharide (G1-4A arabinogalactan polysaccharide) derived from *T. cordifolia* was found to stimulate murine macrophages leading to up-regulation of IL-2, IFN-γ, IL-4, and IL-1β in vitro [40]. Therefore, these results are indicative of the immunomodulatory potential of this herb in chickens, through alteration of the expressions of immune response cytokines. Recent studies have also shown the immunomodulatory potential of *T. cordifolia* stem hydro-alcoholic extract in chickens against a 2.4-dinitrofluorobenzene (DNFB) skin sensitization test, as seen in the form of increases in skin thickness [41]. *T. cordifolia* extract supplementation in chicks has also shown protection against *Escherichia coli* infection by increasing the *E. coli* specific antibody titer and lymphocyte proliferation response [42]. It has been found that G1-4A, an arabinogalactan polysaccharide from the stem of *T. cordifolia,* responsible for its immunomodulatory potential, acts by activating the B cells polyclonally, via an increase in CD69 expression in lymphocytes. TLR4 on B lymphocytes and macrophages acts as a receptor for G1-4A polysaccharide, activating these immune cells via TLR4/MyD88 dependent manner [40,43]. Similarly, G1-4A leads to enhanced antigen presentation from dendritic cells, and further activation of cytotoxic T cells [44]. Apart from that, there is one more component identified from *T. cordifolia* stem extract, termed immunomodulatory protein (single chain acidified protein, 25 kDa) which is reported to possess lymphoproliferative and macrophage stimulating properties [23].

TLRs are the best characterized component of the innate immune system, and have been reported in wide range of species, from arthropods to humans. Microbes stimulate TLRs with their PAMPs which in turn activates both the innate and acquired immune responses [45]. The signals generated out of stimulating these TLRs induce the appropriate types of immune responses, providing protection against relevant pathogens. Based on the immunostimulatory properties of TLR agonists, attempts have been made to employ them as an adjuvant with various vaccine antigens. Chicken TLR agonists have been used successfully as vaccine adjuvants [12,46] and prophylactic agents [13,47]. Previous studies already established the role of CpG ODN (TLR21 agonist) as an immunostimulant in chickens leading to increased protection against various pathogens [48,49]. CpG ODN provides antiviral immunity by enhanced recruitment of macrophages, up-regulation of INF-γ and cluster differentiation of CD8/4+ T lymphocytes [49]. Apart from enhancing cytokine expression, CpG ODN administration has shown an anti-microbial effect by the enrichment of immunological niches (macrophages, CD4, and CD8 T cells population) in various lymphoid organs, such as the spleen and thymus [50].

In this experiment, *T. cordifolia* aqueous extract (group C) and CpG ODN (group D) were evaluated for their prophylactic efficacy against the vvIBDV challenge in chickens at 4 weeks of age. Moreover, their combined effect to protect against the vvIBDV challenge (group E) was also evaluated. Protection was evaluated based on the cellular immune response, humoral response, histopathological, and the protection study. Cellular immune response was evaluated through the quantitative real-time PCR based expression of cytokines (IFN-γ, IL-2) in chicken PBMCs and bursa of Fabricius. Expression levels of IL-4 and IL-1β was also assessed in chicken PBMCs and bursa of Fabricius using qRT-PCR. Humoral immune response was assessed using an ELISA kit for estimating the antibody titer.

The results of the current study indicated highest expression levels of IL-2, IFN-γ, IL-4, and IL-1β in the PBMCs and bursa of Fabricius in group E, followed by group D, and then group C. This indicates that the three groups treated with immunomodulatory agent (either alone or in combination) showed more expression of cytokines under study than the positive virus control (group B) at both 7 DPC and 14 DPC. Significant increases in the expression levels of IL-2 and IFN-γ cytokines in group E point to the additive influence of CpG ODN and *T. cordifolia* extract in stimulating PBMCs and immune cells in the bursa of Fabricius towards a Th1 response, thereby stimulating a cell mediated immune response in a chicken, when challenged with vvIBDV. IL-2 is expressed exclusively by T-lymphocytes, and it promotes the growth of T-lymphocytes, thereby enhancing cell mediated immunity [51]. IFN-γ is a type 2 interferon and a hallmark cytokine for T_H_1 cells [52], producing pleiotropic effects on immune cells; viz., antiviral activity, stimulation of macrophages and natural killer cells, and increased expression of major histocompatibility antigens [53]. Thus, induction of IFN-γ by *T. cordifolia* aqueous extract and TLR 21 agonist suggests that they enhance the ability of the immune system to combat various intracellular pathogens in chickens, which is biased towards Th1 response. This biased Th1 immune response is essential to combat intracellular pathogens such as viruses [54]. Since stimulation of intra-bursal T lymphocytes plays an important role in clearing viral infection and promoting recovery from infection [55], the ability of CpG ODN and *T. cordifolia* aqueous extract in inducing T_H_1 responses, as observed in the present study, suggests the mechanism of their protective effects against vvIBDV in the treated chickens.

The immune system is regulated between T_H_1 and T_H_2 responses and the polarization of the responses is largely based on antigen-specific T_H_ cells. T_H_1 cells drive cell-mediated, inflammatory responses, while T_H_2 cells drive responses against helminthic worms, extracellular bacteria, and some viral infections. T_H_1 cells typically produce IFN-γ, while T_H_2 cells typically produce IL-4 [56]. IL-4 is a pleiotropic lymphokine produced by Th2 cells which plays an important role in the immune system [57]. MHC II is an important molecule for the antigen presentation of B cells. IL-4 enhances expression of MHC II and IL-4 receptors on B lymphocytes and these receptors have an important role in B cell function. Chicken bone marrow cells cultured in the presence of recombinant chicken IL-4 get transformed into dendritic cells and express increased MHC II [58]. MHC II is required to present exogenous antigens processed by endocytic pathway and promote T_H_2 mediated immunity [59]. IL4 expression in this study shows a stimulatory effect of immune modulatory supplements over IL4 cytokine (as evidenced from its significant increase in groups E, D, and C), thus indicating activated B lymphocyte responses and humoral immunity against vvIBDV challenge. Thus, the findings of this study suggest that TLR 21 agonist and *T. cordifolia* aqueous extract can induce a mixed and more balanced T_H_1 and T_H_2 response which is vital to protect a host against distinct types of microbes, such as viruses and worms.

Besides the above described adaptive responses, the influences of CpG ODN and *T. cordifolia* aqueous extract in stimulating macrophages could be evaluated from the expression of the pro-inflammatory cytokine, chicken IL-1β, which is a mammalian homolog produced from avian macrophages [60]. IL-1β acts as a chemotactic factor for macrophages at the site of injury where they differentiate into tissue macrophages. These tissue macrophages carry out various functions, such as the release of nitric oxide (NO) which is needed for viral destruction, phagocytosis of antigens, and antigen processing and presentation [60].

Humoral immune response stimulation in vvIBDV challenged birds was evaluated through estimating the antibody titer using an ELISA kit. The antibody titer was found to be highest in group E, followed by group D, and then group C. The immunomodulatory agent treated groups showed significantly higher IBDV specific antibody titers than the positive virus control group at both 7 DPC and 14 DPC. This correlates with the level of IL-4 levels estimated by qRT-PCR. Anti-IBDV antibody plays an important role in protecting the chicken against IBDV infection [61].

Bursal damage, as seen histopathologically, was found to be least in group E, followed by groups D and C, respectively. Positive control (group B) showed extensive bursal damage. The least bursal damage in group E might be due to the highest virus clearance in their bursa. This clearance of virus is due to the stimulation of the cell mediated immune response as indicated by high IFN-γ and IL-2 expression levels in both their bursa and PBMCs [62].

Moreover, this study provides evidence that the challenging of SPF chickens with vvIBDV in group E induced an obvious protective immune response to avoid an otherwise lethal outcome. The protection of 100% from vvIBD virus was achieved in the group E which received CpG ODN along with *T. cordifolia* aqueous extract. In groups C and D, 50% and 80% protection was observed, respectively. In the positive virus control (group B), 100% mortality was observed during the observation period. These birds showed characteristics signs of IBDV infection, such as depression, drowsiness, inability to move, ruffled feathers, and severe prostration. On necropsy examination of birds in group B, characteristics of IBDV lesions were found, such as hemorrhages in the thigh muscles, splenomegaly, and swollen and hemorrhagic bursae. The bursal folds were extensively hemorrhagic and contained variable amounts of blood clotting.

Further, immunosuppression, which is the most serious impact of IBDV infection, was found to be diluted in immunomodulatory agent treated groups, with the highest dilution in group E, followed by group D, and then group C. This finding is significant, since vaccination with live vaccines leaves birds protected to some extent, but birds have lowered immunity, and further vaccination against other diseases fails, leaving them vulnerable to various pathogens.

Antibody response was highest after IBDV vaccine in group E, followed by group D, and then group C. The immunomodulatory agent treated groups showed significantly higher IBDV specific antibody titers than positive vaccine control group at 7 DPV, 14 DPV, 21 DPV, and 28 DPV. Thus, both the immunomodulatory agents possess adjuvant activity with IBDV vaccine. Such adjuvants with balanced stimulation of the immune response and with less adverse effects are highly desirable to achieve efficient protection against infectious diseases.

## 5. Conclusions

To conclude, we suggest that *T. cordifolia* and CpG ODN be used as prophylactic agents, and as adjuvants in poultry for viral diseases. They both have immunomodulatory potential via the TLR mediated pathway. There is evidence for using a combination of TLR agonist as an adjuvant in vaccines for a better immune response [63]. However, TLR agonists are costly compared with herbal preparations. If one of the synthetic TLR agonists acting via one TLR can be substituted with the herb acting through the same TLR pathway, then the cost of the vaccine can be further reduced. To further understand the mechanism of synergy between this herb and CpG ODN, studies must be designed to evaluate various intermediate adaptor molecules and their interaction in immune cells. Other herbal preparations and TLR agonists can also be evaluated for their synergistic effects of stimulating the immune response against various infectious diseases. Evaluation of herbs and their extracts to modulate the innate immune response will be helpful, as they are cheap and easily available to farmers for use in the form of immunomodulatory agents, to protect the poultry flock against a variety of infectious diseases.

## Figures and Tables

**Table 1:** Details of the oligonucleotide primer sequence used for chicken specific genes in qReal-time PCR.

Name of the Gene	Primer sequence (5′ → 3′)	Reference
IL-2	TTCTGGGACCACTGTATGCTCTT TACCGACAAAGTGAGAATCAATCAG	AF000631.1-Gene bank accession no.
IL-4	GGAGAGCATCCGGATAGTGA TGACGCATGTTGAGGAAGAG	[28]
IFN-γ	AAGTCAAAGCCGCACATCAAAC CTGGATTCTCAAGTCGTTCATCG	X99774.1-Gene bank accession no.
β-actin	TATGTGCAAGGCCGGTTTC TGTCTTTCTGGCCCATACCAA	[29]
IL-1β	GGATTCTGAGCACACCACAGT TCTGGTTGATGTCGAAGATGTC	[28]
VVIBD-VP2 gene	CCTGGCTCAATTGTGGGTGCTCA GGCGTTTATGGTTCCGTTTAGTGC	Custom designed

**Table 2:** Quantitative real time PCR analysis of immune response genes (expressed as fold change in the immune response gene transcripts) in chicken PBMCs after *T. cordifolia* treatment at 10 mL/bird/day from day old to 4 week of age.

S. number	Group	1L-2	IFN-γ	IL-4	IL-1β
1.	Control	1 ^a^ ± 0.00	1 ^a^ ± 0.00	1 ^a^ ± 0.00	1 ^a^ ± 0.00
2.	*T. cordifolia*	5.657 ^b^ ± 0.1663	15.15 ^b^ ± 0.2288	8.458 ^b^ ± 0.277	13.09 ^b^ ± 0.2681

^a,b^ Means bearing different superscripts column-wise differ significantly (*p* < 0.05).

**Table 3:** Quantitative real-time PCR analysis of IL-2 expression in PBMCs of chickens challenged with very virulent, infections bursal disease virus (vvIBDV) at different days post challenge (DPC).

S. number	Group	7 DPC	14 DPC
1.	Negative control (Group A)	1 ^a^ ± 0.00	1 ^a^ ± 0.00
2.	Positive control (Group B)	3.977 ^b^ ± 0.200	2.957 ^b^ ± 0.210
3.	*T. cordifolia* + virus challenge (Group C)	6.350 ^c^ ± 0.132	5.740 ^c^ ± 0.231
4.	CpG ODN + virus challenge (Group D)	8.068 ^d^ ± 0.207	7.462 ^d^ ± 0.507
5.	*T. cordifolia* + CpG ODN + challenge (Group E)	10.840 ^e^ ± 0.232	10.620 ^e^ ± 0.723

^a–e^ Means bearing different superscripts column-wise differ significantly (*p* < 0.05). The results are expressed as fold changes to the immune response gene transcripts relative to the control group.

**Table 4:** Quantitative real time PCR analysis of IFN-γ expression in PBMCs of chickens challenged with vvIBDV at different DPC.

S. number	Group	7 DPC	14 DPC
1.	Negative control (Group A)	1 ^a^ ± 0.00	1 ^a^ ± 0.00
2.	Positive control (Group B)	8.570 ^b^ ± 0.303	8.480 ^b^ ± 0.376
3.	*T. cordifolia* + virus challenge (Group C)	13.953 ^c^ ± 0.224	15.892 ^c^ ± 0.509
4.	CpG ODN + virus challenge (Group D)	18.672 ^d^ ± 0.490	19.973 ^d^ ± 0.094
5.	*T. cordifolia* + CpG ODN + challenge (Group E)	25.702 ^e^ ± 0.328	26.982 ^e^ ± 0.345

^a–e^ Means bearing different superscripts column-wise differ significantly (*p* < 0.05). The results are expressed as fold changes to the immune response gene transcripts relative to the control group.

**Table 5:** Quantitative real time PCR analysis of IL-4 expression in PBMCs of chickens challenged with vvIBDV at different DPC.

S. number	Group	7 DPC	14 DPC
1.	Negative control (Group A)	1 ^a^ ± 0.00	1 ^a^ ± 0.00
2.	Positive control (Group B)	8.288 ^b^ ± 0.294	7.447 ^b^ ± 0.172
3.	*T. cordifolia* + virus challenge (Group C)	11.870 ^c^ ± 0.315	12.017 ^c^ ± 0.364
4.	CpG ODN + virus challenge (Group D)	15.265 ^d^ ± 0.162	15.397 ^d^ ± 0.293
5.	*T. cordifolia* + CpG ODN + challenge (Group E)	19.400 ^e^ ± 0.376	20.468 ^e^ ± 0.207

^a–e^ Means bearing different superscripts column-wise differ significantly (*p* < 0.05). The results are expressed as fold changes to the immune response gene transcripts relative to the control group.

**Table 6:** Quantitative real time PCR analysis of IL-1β expression in PBMCs of chickens challenged with vvIBDV at different DPC.

S. number	Group	7 DPC	14 DPC
1.	Negative control (Group A)	1 ^a^ ± 0.00	1 ^a^ ± 0.00
2.	Positive control (Group B)	5.077 ^b^ ± 0.128	4.992 ^b^ ± 0.276
3.	*T. cordifolia* + virus challenge (Group C)	9.650 ^c^ ± 0.240	8.987 ^c^ ± 0.390
4.	CpG ODN + virus challenge (Group D)	11.987 ^d^ ± 0.243	11.660 ^d^ ± 0.123
5.	*T. cordifolia* + CpG ODN + challenge (Group E)	15.628 ^e^ ± 0.444	15.247 ^e^ ± 0.169

^a–e^ Means bearing different superscripts column-wise differ significantly (*p* < 0.05). The results are expressed as fold changes to the immune response gene transcripts relative to the control group.

**Table 7:** Quantitative real time PCR analysis of IL-2 expression in the bursa of Fabricius of chickens challenged with vvIBDV at different DPC.

S. number	Group	7 DPC	14 DPC
1.	Negative control (Group A)	1 ^a^ ± 0.00	1 ^a^ ± 0.00
2.	Positive control (Group B)	7.838 ^b^ ± 0.104	7.840 ^b^ ± 0.127
3.	*T. cordifolia* + virus challenge (Group C)	12.912 ^c^ ± 0.524	12.148 ^c^ ± 0.188
4.	CpG ODN + virus challenge (Group D)	17.397 ^d^ ± 0.232	14.797 ^d^ ± 0.189
5.	*T. cordifolia* + CpG ODN + challenge (Group E)	27.550 ^e^ ± 0.363	27.370 ^e^ ± 0.496

^a–e^ Means bearing different superscripts column-wise differ significantly (*p* < 0.05). The results are expressed as fold changes to the immune response gene transcripts relative to the control group.

**Table 8:** Quantitative real time PCR analysis of IFN-γ expression in the bursa of Fabricius of chickens challenged with vvIBDV at different DPC.

S. number	Group	7 DPC	14 DPC
1.	Negative control (Group A)	1 ^a^ ± 0.00	1 ^a^ ± 0.00
2.	Positive control (Group B)	13.322 ^b^ ± 0.154	13.137 ^b^ ± 0.153
3.	*T. cordifolia* + virus challenge (Group C)	22.490 ^c^ ± 0.407	23.758 ^c^ ± 0.766
4.	CpG ODN + virus challenge (Group D)	31.348 ^d^ ± 0.298	31.232 ^d^ ± 0.659
5.	*T. cordifolia* + CpG ODN + challenge (Group E)	40.892 ^e^ ± 0.446	39.693 ^e^ ± 2.031

^a–e^ Means bearing different superscripts column-wise differ significantly (*p* < 0.05). The results are expressed as fold changes to the immune response gene transcripts relative to the control group.

**Table 9:** Quantitative real time PCR analysis of IL-4 expression in the bursa of Fabricius of chickens challenged with vvIBDV at different DPC.

S. number	Group	7 DPC	14 DPC
1.	Negative control (Group A)	1 ^a^ ± 0.00	1 ^a^ ± 0.00
2.	Positive control (Group B)	1.962 ^b^ ± 0.145	1.838 ^b^ ± 0.178
3.	*T. cordifolia* + virus challenge (Group C)	2.600 ^c^ ± 0.169	2.788 ^c^ ± 0.056
4.	CpG ODN + virus challenge (Group D)	3.712 ^d^ ± 0.172	3.310 ^d^ ± 0.195
5.	*T. cordifolia* + CpG ODN + challenge (Group E)	5.842 ^e^ ± 0.272	5.663 ^e^ ± 0.211

^a–e^ Means bearing different superscripts column-wise differ significantly (*p* < 0.05). The result is expressed as fold change in the immune response gene transcripts relative to control group.

**Table 10:** Quantitative real time PCR analysis of IL-1β expression in the bursa of Fabricius of chickens challenged with vvIBDV at different DPC.

S. number	Group	7 DPC	14 DPC
1.	Negative control (Group A)	1 ^a^ ± 0.00	1 ^a^ ± 0.00
2.	Positive control (Group B)	2.222 ^b^ ± 0.055	2.165 ^b^ ± 0.157
3.	*T. cordifolia* + virus challenge (Group C)	3.427 ^c^ ± 0.075	3.360 ^c^ ± 0.092
4.	CpG ODN + virus challenge (Group D)	5.543 ^d^ ± 0.128	5.345 ^d^ ± 0.133
5.	*T. cordifolia* + CpG ODN + challenge (Group E)	7.498 ^e^ ± 0.054	7.275 ^e^ ± 0.413

^a–e^ Means bearing different superscripts column-wise differ significantly (*p* < 0.05). The results are expressed as fold changes to the immune response gene transcripts relative to the control group.

**Table 11:** Serum antibody titer (mean ± SE of antilog of Log_10_ titer) in chickens challenged with vvIBDV at different DPC by ELISA kit method.

S. number	Group	Pre-Challenge	7 DPC	14 DPC
1.	Negative control (Group A)	21.345 ^a^ ± 0.767	8.070 ^a^ ± 1.702	25.658 ^a^ ± 1.397
2.	Positive control (Group B)	8.773 ^b^ ± 0.659	569.689 ^b^ ± 15.910	737.271 ^b^ ± 6.345
3.	*T. cordifolia* + virus challenge (Group C)	7.048 ^c^ ± 0.400	699.968 ^c^ ± 17.517	809.135 ^c^ ± 3.712
4.	CpG ODN + virus challenge (Group D)	135.942 ^d^ ± 2.754	871.597 ^d^ ± 7.625	1776.421 ^d^ ± 7.847
5.	*T. cordifolia* + CpG ODN + challenge (Group E)	35.355 ^e^ ± 2.604	1152.477 ^e^ ± 8.040	2115.643 ^e^ ± 18.462

^a–e^ Means bearing different superscripts column-wise differ significantly (*p* < 0.05).

**Table 12:** Protection observed in different vvIBDV challenge groups.

S. Number	Group	Total Number of Birds Challenged	Total Number of Birds Live	Total Number of Birds Dead	Percentage of Live Birds
1.	Negative control (Group A)	---	20	---	100%
2.	Positive control (Group B)	20	---	20	0%
3.	*T. cordifolia* + virus challenge (Group C)	20	10	10	50%
4.	CpG ODN + virus challenge (Group D)	20	16	4	80%
5.	*T. cordifolia* + CpG ODN + challenge (Group E)	20	20	---	100%

**Table 13:** Viral load (mean log_10_ copy number ± SE) in the bursa of Fabricius (gene copy number by micrograms of total RNA) of chickens in different groups challenged with vvIBDV at 4 DPC.

S. Number	Group	4 DPC
1.	Negative control (Group A)	8.840 ^a^ ± 0.180
2.	*T. cordifolia* + virus challenge (Group C)	5.910 ^b^ ± 0.262
3.	CpG ODN + virus challenge (Group D)	3.977 ^c^ ± 0.222
4.	*T. cordifolia* + CpG ODN + challenge (Group E)	2.647 ^d^ ± 0.277

^a–d^ Means bearing different superscripts column-wise differ significantly (*p* < 0.05).

**Table 14:** Hemagglutination inhibition (HI) antibody titer (mean log_2_ HI titer ± SE) against the New Castle disease vaccine in *T. cordifolia* and/or CpG treated and vvIBDV challenged WL chicken.

S. Number	Group	1 Wk Post NDV Vaccination	2 Wk Post NDV Vaccination	3 Wk Post NDV Vaccination
1.	Negative control (Group A)	1.314 ^a^ ± 0.014	3.684 ^a^ ± 0.029	6.788 ^a^ ± 0.061
2.	*T. cordifolia* + virus challenge (Group C)	1.116 ^b^ ± 0.072	2.668 ^b^ ± 0.143	5.518 ^b^ ± 0.157
3.	CpG ODN + virus challenge (Group D)	1.204 ^c^ ± 0.061	2.820 ^c^ ± 0.051	5.908 ^c^ ± 0.064
4.	*T. cordifolia* + CpG ODN + challenge (Group E)	1.298 ^d^ ± 0.018	3.640 ^d^ ± 0.034	6.750 ^d^ ± 0.077

^a–d^ Means bearing different superscripts column-wise differ significantly (*p* < 0.001).

**Table 15:** Serum antibody titers (mean ± SE of antilog of Log_10_ titer) in chickens vaccinated with IBDV vaccine at different DPV by ELISA kit method.

S. Number	Group	Pre-Vaccination	7 DPV	14 DPV	21 DPV	28 DPV
1.	Negative control	127.367 ^a^±1.735	16.476 ^a^±0.895	18.914 ^a^±0.087	23.683 ^a^±0.591	57.440 ^a^±2.618
2.	Positive control	5.545 ^b^ ± 0.109	404.624 ^b^ ± 1.797	466.811 ^b^ ± 6.808	576.626 ^b^ ± 8.290	728.446 ^b^ ± 3.561
3.	*T. cordifolia* + vaccine	6.340 ^c^ ± 0.195	525.834 ^c^ ± 3.913	602.523 ^c^ ± 5.427	716.618 ^c^ ± 11.656	944.575 ^c^ ± 12.222
4.	CpG ODN + vaccine	19.897 ^d^ ± 0.190	587.897 ^d^ ± 11.084	717.898 ^d^ ± 11.729	1217.303 ^d^ ± 18.584	1857.785 ^d^ ± 4.094
5.	*T. cordifolia* + CpG ODN + vaccine	124.547 ^e^ ± 1.614	784.827 ^e^ ± 12.559	979.217 ^e^ ± 9.894	2020.827 ^e^ ± 20.893	2509.359 ^e^ ± 12.774

^a–e^ Means bearing different superscripts column-wise differ significantly (*p* < 0.001).
